# Supplement Intake in Recreational Vegan, Vegetarian, and Omnivorous Endurance Runners—Results from the NURMI Study (Step 2)

**DOI:** 10.3390/nu13082741

**Published:** 2021-08-10

**Authors:** Katharina Wirnitzer, Mohamad Motevalli, Derrick R. Tanous, Martina Gregori, Gerold Wirnitzer, Claus Leitzmann, Lee Hill, Thomas Rosemann, Beat Knechtle

**Affiliations:** 1 Department of Subject Didactics and Educational Research and Development, University College of Teacher Education Tyrol, 6020 Innsbruck, Austria; katharina.wirnitzer@ph-tirol.ac.at; 2 Department of Sport Science, University of Innsbruck, 6020 Innsbruck, Austria; mohamad_motevali@yahoo.com (M.M.); derrick.tanous@student.uibk.ac.at (D.R.T.); 3 Life and Health Science Cluster Tirol, Subcluster Health/Medicine/Psychology, 6020 Innsbruck, Austria; 4 Research Center Medical Humanities, Leopold-Franzens University of Innsbruck, 6020 Innsbruck, Austria; 5 Faculty of Physical Education and Sports Sciences, Ferdowsi University of Mashhad, Mashhad 9177948974, Iran; 6 Department of Nutritional Sciences, University of Vienna, Althanstrasse 14, 1090 Vienna, Austria; a00805153@unet.univie.ac.at; 7 Adventurev & Change2v, 6135 Stans, Austria; gerold@wirnitzer.at; 8 Institute of Nutrition, University of Gießen, 35390 Gießen, Germany; claus@leitzmann-giessen.de; 9 Department of Pediatrics, Division of Gastroenterology and Nutrition, McMaster University, Hamilton, ON L8N 3Z5, Canada; hilll14@mcmaster.ca; 10 Institute of Primary Care, University of Zurich, 8091 Zurich, Switzerland; thomas.rosemann@usz.ch; 11 Medbase St. Gallen Am Vadianplatz, 9001 St. Gallen, Switzerland

**Keywords:** nutrition, diet, plant-based, supplement, ergogenic aids, supplementation, marathon, half marathon, running, athlete

## Abstract

Nutrient deficiency is a common cause of underperformance in endurance athletes, and supplement intake is frequently considered compensatory for vegan and vegetarian athletes specifically. This study aimed to investigate the patterns of supplement intake among vegan, vegetarian, and omnivorous distance (>10 km) runners and its association with age, sex, and race distance. From a total of 317 runners who participated in an online survey, 220 distance runners (mean age: 38.5 years; mean BMI: 21.75 kg/m^2^) were selected for the final sample after data clearance and assigned to 100 omnivores, 40 vegetarians, or 80 vegans. Sociodemographic information, racing experience, and patterns of supplement intake, including type, frequency, dosage, etc., were collected using a questionnaire. Macronutrient intake was assessed using a food frequency questionnaire. ANOVA and logistic regression were used for data analysis. The prevalence of supplement intake was 51% for total runners and 72% among vegan runners. Age, sex, and race distance had no significant effect on the type of supplement intake (*p* > 0.05). Compared to omnivores and vegetarians, vegan runners reported consuming more vitamin (but not carbohydrate/protein or mineral) supplements (*p* < 0.05). Vitamin B_12_, magnesium, and multivitamin had the most prevalent use amongst micronutrient supplements. This study points to a central role for supplementary nutritional strategies in different groups of distance runners. The present findings may help future investigations by design to identify specific requirements of endurance runners when adhering to specific kinds of diet particularly plant-based diets.

## 1. Introduction

Plant-based diets are increasingly being followed more for various reasons, including health, performance, ethical, and environmental concerns [1,2]. While the bioavailability of vitamin B_12_, vitamin D, protein, calcium, iron, iodine, and zinc has been reported to be lower in many consuming plant-based diets compared to diets containing foods from animal sources [2,3], it has been well-documented that plant-based diets typically have higher amounts of carbohydrates and antioxidants [2,4], which can be advantageous for endurance performance [1]. Despite the well-documented beneficial aspects of plant-based diets among different populations, including athletes, there are still some concerns discussed regarding nutrient adequacy of vegan and vegetarian diets for supporting athletic performance, particularly for those who engage in endurance activities [5]. 

Irrespective of diet type, there is scientific evidence that indicates endurance runners may not be consuming sufficient nutrients through daily foods to support their athletic needs [6], and this concern might be more critical for endurance athletes who are generally at a higher risk of low energy availability [7] and/or follow specific kinds of diets, especially when the diet is not planned appropriately [5]. In such a situation, paying careful attention to the optimal intake of supplemental macronutrients and micronutrients has been highly recommended to both vegan and vegetarian athletes [5]. Inadequacy of nutrient intake may be due to avoiding gastrointestinal distress, the inability to manage meal frequencies, and poor nutritional knowledge of food choice such as adhering to non-scientific regimens (e.g., low-carbohydrate diet) in particular [8,9]. Additionally, competition-related factors (e.g., suppressed appetite and increased anxiety) may limit nutrient intake during consecutive days of pre- and mid-events [8]. While the important crosstalk between diet, metabolic pathways, and VO_2_max has been well-established in the literature [10], current evidence shows that both elite [11] and recreational [12] endurance athletes are at risk of consuming inadequate dietary intake to meet the requirements of daily training and health. In practice, however, if the well-recognized dietary needs of an endurance athlete could be translated to a well-planned diet, evidence indicates that even ultra-endurance challenges, such as a mountain bike race, can be successfully and healthfully completed by implementing a vegan diet [13].

As a commonly used approach amongst many athletes, dietary supplements are frequently consumed to fulfill the nutrient needs and enhance physical performance and/or appearance [14]. Although the beneficial effects of many supplements on the promotion of health, prevention of chronic disease, and enhancement of athletic performance remain unclear [14,15], it is well-established that these products significantly contribute to the nutrient requirements of athletes [15,16]. Recreational endurance athletes may have different choices of dietary supplements, which could be associated with their differing goals of engaging in training and competition, compared to elite athletes [17]. In general, careful consideration for a balance between potential benefits (e.g., contribution to health and performance) and risks (e.g., adverse health effects, distraction, supplement contamination) has been recommended to endurance athletes when using dietary supplements [17].

Although numerous studies demonstrate that the nutrient deficiencies in plant-based diets are generally due to poor planning of regime [1,18], evidence indicates that many vegan and vegetarian athletes frequently use nutritional supplements [5,19], particularly the scientifically emphasized micronutrients such as vitamin B_12_, vitamin D, and iron [5,20]. It has been reported that a considerable portion of the daily micronutrient needs of vegan endurance runners comes from micronutrient supplements [20]. This has led to the majority of vegan (but only half of the vegetarian and the omnivorous) endurance runners meeting their nutritional recommendations for many micronutrients [20]. 

To date, few studies have assessed the nutritional patterns and supplement intake in vegan and vegetarian athletes [21,22]. Despite these issues, and considering the importance of nutritional demands of vegan and vegetarian athletes, patterns of supplement intake among vegan and vegetarian athletes are not yet clear, and current studies investigating the relationship between plant-based diets and nutrient intakes did not discriminate between vegans and vegetarians [23] or different running distances [19,20]. Considering the rising number of athletes adhering to plant-based diets [4,5], and given the limited data investigating the nutritional concerns of vegan and vegetarian endurance athletes, the purpose of this cross-sectional study was to provide a comprehensive profile of supplement intake among a partially large number of vegan, vegetarian, and omnivorous endurance runners, and assess its association with age, sex, and race distance.

## 2. Materials and Methods

### 2.1. Study Design and Ethical Approval

The present study was designed as descriptive and cross-sectional and is part of the NURMI (Nutrition and Running High Mileage) study step 2 [24]. Endurance runners in the NURMI study were mainly recruited from German-speaking countries, including Austria, Germany, and Switzerland. The method described subsequently had been introduced in detail previously [21,24,25]. The study protocol was approved by the ethics board of St. Gallen, Switzerland, on 6 May 2015 (EKSG 14/145), with a trial registration number ISRCTN73074080.

### 2.2. Participants and Experimental Approach

Participants completed an online survey within step 2 of the NURMI study, which was available in German and English at www.nurmi-study.com, accessed on 6 August 2021. Before completing the questionnaire, participants were provided with a written description of the procedures and gave their informed consent to participate in the study. The questionnaire consisted of several parts, e.g., running training and racing behavior, quality of life, food frequency [26], as well as physical and dietary information, including regular supplement intake (frequency per week, type, amount, specification for nutrient) and estimated percentage of macronutrients. For successful participation in the NURMI study step 2, the following inclusion criteria were required: (1) written informed consent, (2) at least 18 years of age, (3) questionnaire step 2 completed, (4) having a BMI ≥ 30 kg/m^2^ [27,28], and (5) successful participation in a running event of at least the half-marathon distance in the past two years. However, a total of 79 10-km runners who had not successfully participated in either a half-marathon or a marathon also provided accurate and useful answers with plenty of high-quality data. To avoid an irreversible loss of these valuable data sets, 10-km runners who met the inclusion criteria were included in the study population.

### 2.3. Data Clearance

**Figure 1:**
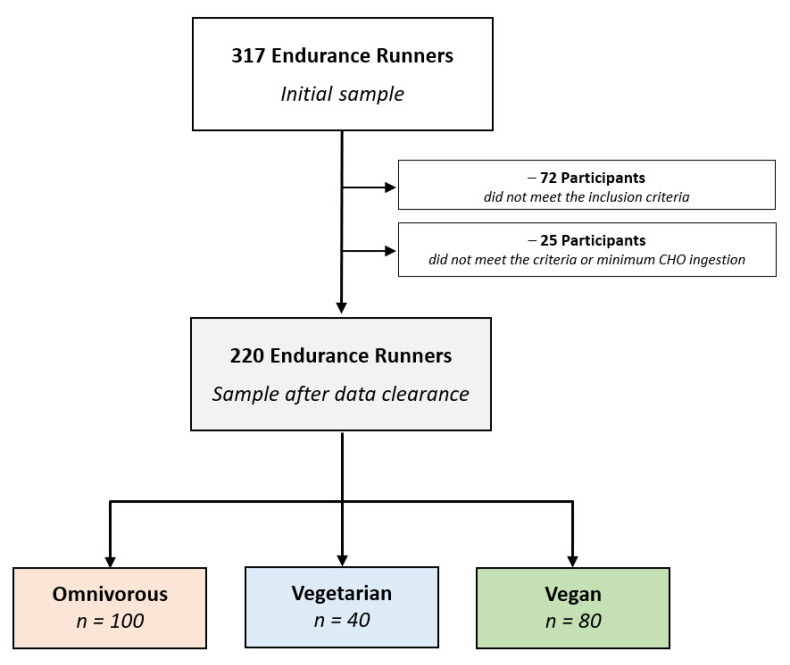
Enrollment and categorization of participants by dietary subgroups. CHO—carbohydrates.

From a total number of 317 endurance runners, the initial sample of the survey, 72 participants who did not meet the aforementioned general inclusion criteria (e.g., those who did not answer all essential questions including diet type, age, sex, and race distance) were excluded from the study. As a consequence of the specific exclusion criteria for the present study, an additional 25 runners were recognized for consuming less than 50% carbohydrates in their dietary intake, which is lower than the minimum amount of recommended level [1,29], and were excluded from the analysis, due to the avoidance of contradictory data in supplement intake [29]. Moreover, the diet types of nine runners were modified from vegan or vegetarian to omnivores via applying control questions through the questionnaire. For the purpose of data analysis, participants were assigned into three dietary subgroups, including omnivorous diet (or Western diet, defined as diets with no restriction on any kind of food), vegetarian diet (defined as diets devoid of all flesh foods but include egg and/or dairy products), and vegan diet (or plant-based diet, defined as diets devoid of all foods and products from animal sources including meat and processed meat, fish, shellfish and other so-called seafood, animal fats/oils, milk and dairy, eggs, honey, and related byproducts) [2]. Figure 1 shows the participants’ enrollment within the current study.

### 2.4. Measures

The participants were asked to report their regular supplement intake, in addition to food frequency (unpublished data from our laboratory based on the food frequency questionnaire of the “German Health Interview and Examination Survey for Adults (DEGS)” with friendly permission of the Robert Koch Institute, Berlin, Germany) [30]. Macronutrient intake of endurance runners was described by the self-reported percentage of daily calories from carbohydrate, protein, and fat. Supplement intake was described by the following items: regular intake; frequency; kind of supplement: carbohydrate/protein (whether separately or in conjugation), minerals, vitamins; brand of supplement (with nutrient that provides the main contribution); amount; other substances; and was linked to age, sex, diet type, and running distance.

The latent factors “running experience” (“age.first.running event”, “age.run”, “age.first.half-marathon”, “age.first.marathon”) and “racing experience” (“years.running”, “completed.half-marathon.number”, “completed.marathon.number”) were derived by using both pooled items defined by specific items based on manifest variables. Since the running experience (e.g., number of years of active running, age of first running event, number of completed races) depends on age, the respective items were operationalized with age (e.g., age-related years of running, age-related number of completed races over half-marathon distance). With this, the respective items (e.g., age-related start of running, completion of first marathon race) were centered by the median and were z-transformed to a new scale by summarizing the respective items (e.g., years of running, number of completed races over specific distances). Based on this, the values were categorized considering the two latent factors “running experience” and “racing experience” as low (values below –1), medium (values ranging from −1 to +1), and high (values higher +1).

### 2.5. Statistical Analysis

The statistical software R version 4.0.0 (R Foundation for Statistical Computing, Vienna, Austria) was used to perform all statistical analyses. Exploratory analysis was performed by descriptive statistics (median and interquartile range (IQR), mean and standard deviation (SD)). Significant differences between the types of supplement intake and dietary subgroups, age, sex, and race distance were calculated by ANOVA. Logistic regression analysis (95% confidence interval (95%-CI)) was used to determine the effect size of the variables (age, sex, diet type, race distance) on intake from kind of supplements (macronutrients, minerals, vitamins) and is displayed as effect plots. Chi-square test (χ^2^, nominal scale) was used to examine the association between dietary subgroups and age, sex, race distance. Kruskal-Wallis tests (ordinal and metric scale) were approximated by using the t or F distributions or using ordinary least squares and standard errors (SE), and R^2^. Logistic regression analysis (95% confidence interval (95%-CI)) was used to determine the effect size of the variables (age, sex, race distance) on intake from kind of supplements (macronutrients, minerals, vitamins) and is displayed as effect plots (95% confidence interval (95%-CI)). The level of statistical significance was set at *p* ≤ 0.05.

## 3. Results

From a total number of 317 endurance runners, 220 participants (127 women and 93 men) with a mean age of 38.5 (IQR 18.0) years remained for statistical analysis after a multi-stage data clearance. Germany, Austria, and Switzerland had the majority of endurance runners with 161, 39, and 11 participants, respectively, while 9 participants were from other countries (including Belgium, Brazil, Canada, Italy, Luxemburg, Netherlands, Poland, Spain, and the UK). Based on their race distance, there were a total of 79 10-km runners, 84 half marathoners, and 57 (ultra-)marathoners.

In the descriptive characteristics, there was a significant difference in absolute values of body weight (F_(2, 217)_ = 6.99, *p* = 0.001) and BMI (F_(2, 217)_ = 7.32, *p* < 0.001) but not in body height. In addition, both vegans and vegetarians were significantly younger (F_(2, 217)_ = 3.17, *p* = 0.041) than omnivores. Moreover, there was a significant difference between sex and diet type (χ^2^_(2)_ = 8.71, *p* = 0.013), with the majority of vegans and vegetarians being female. No significant association was found between the kind of diet and academic qualification, marital status, country of residence, and race distance (Table 1).

While 51% of total participants reported consuming supplements regularly, the intakes of carbohydrate (CHO)/protein, minerals, vitamins, and other supplements were 20%, 33%, 42%, and 6%, respectively. No difference between vegans (20%), vegetarians (18%), and omnivores (20%) was found regarding the consumption of CHO/protein supplements. The prevalence of mineral supplement intake was lower among vegetarians (22%) compared to vegans (33%) and omnivores (36%). It was found that 66% of vegans consumed vitamin supplements, while only 25% of vegetarians and 30% of omnivores reported consuming vitamin supplements. Results from analysis of open questions showed that amongst mineral supplements, magnesium had the most prevalence of use in all three groups of omnivorous (45%), vegetarian (25%), and vegan (18%) runners. Amongst vitamin supplements, while vitamin B_12_ was reported to be more often consumed by vegan (52%) and vegetarian (38%) runners, the consumption of a multivitamin (30%) and vitamin D (18%) was higher than other vitamin supplements in the omnivorous group (Table 2). 

Concerning the frequency of supplement intake, 58% of the participants reported consuming supplements regularly in their daily diet, while the remaining 42% mentioned either less than once per week (6%), 1–2 times per week (15%), 3–4 times per week (12%), or 5–6 times per week (9%) as the frequency of supplement consumption. When comparing the dietary groups, we found that 61% of the omnivores, 36% of the vegetarians, and 62% of the vegans reported consuming a supplement on a daily basis. Analysis of the self-reported distribution of macronutrients showed that 58.2%, 24.5%, and 17.4% of the total energy intake of participants derived from CHO, protein, and fat, respectively. 

Based on the results from ANOVA and the logistic regression analysis, vitamin supplements were shown to have a statistically significant association (χ^2^_(2)_ = 27.38, *p* < 0.001) with the kind of the diet (particularly the greater intake of vitamin supplements by vegan group), and no significant effects were observed for CHO/protein (χ^2^_(2)_ = 0.10, *p* = 0.951) and minerals (χ^2^_(2)_ = 2.65, *p* = 0.266) across vegan, vegetarian, and omnivorous runners (Table 3). 

**Figure 2:**
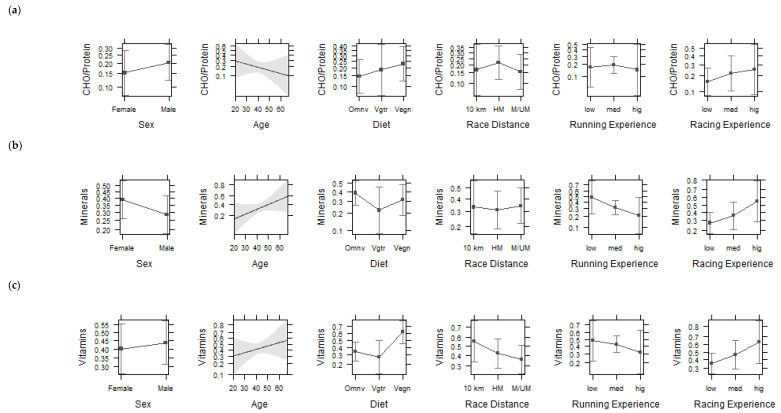
Effect plots with 95% CI for interactions between sex, age, diet type, race distance, running experience, racing experience, and supplement intake from carbohydrates/protein (**a**), minerals (**b**), and vitamins (**c**). CHO—carbohydrates. Omnv—omnivorous. Vgtr—vegetarian. Vegn—vegan. 10 km—10-km. HM—half-marathon. M—Mmarathon/ultra-marathon.

It was found that neither age nor sex has a significant effect on the type of supplement intake in vegan, vegetarian, and omnivorous runners (*p* > 0.05). There was also no significant association between supplement intake and race distance, running experience (low, medium, and high), and racing experience (low, medium, and high) (*p* > 0.05) (Figure 2). No significant effects considering macronutrient intake were observed by sex, age, race distance, running experience, racing experience among dietary groups (*p* > 0.05).

## 4. Discussion

The purpose of this study was to investigate supplement intake among vegan, vegetarian, and omnivorous distance runners and its relation to age, sex, and race distance. As the main finding, we observed a higher prevalence use of vitamin supplements use in vegan endurance runners (66%) compared to vegetarian (25%) and omnivorous (30%) counterparts. Further significant findings were: (1) irrespective of diet type, 51% of the distance runners reported consuming a supplement regularly; (2) vitamin B_12_ and magnesium had the most prevalence for use amongst vitamin and mineral supplements, respectively; (3) there was no association between type of supplement and age, sex, and race distance; and (4) higher CHO and lower protein and fat intakes were observed in macronutrient distribution considering the total daily energy intake of vegans compared to vegetarians and omnivorous endurance runners. Additionally, vegan and vegetarian distance runners had significantly lower body weight and BMI than omnivores that might be associated with the unbalanced sex and age distribution between dietary groups in the present study, as well as a more pronounced health-consciousness of vegetarians but especially vegans compared to omnivores, in general [21].

### 4.1. Diet Type and Supplement Intake

Detailed characteristics of dietary, sports nutrition, and performance-enhancing supplements and the scenarios in which they contribute to the nutritional demands of endurance athletes were presented recently by Maughan et al. [15] and Peeling et al. [31]. In general, performance improvements and meeting nutrient requirements to optimize training demands and recovery are two major goals that persuade endurance runners to use supplements. The present findings showed that 51% of endurance runners consume supplements regularly and that vitamin supplements were the most frequently consumed type (42%) by endurance runners in this study. Results from three European studies performed on 527, 553, and 446 elite athletes showed that the prevalence of supplement intakes was 64%, 62%, and 81%, respectively [32,33,34]. These reports were higher compared to a 40% prevalence in consumption of performance supplements reported by another study on marathoners [35], possibly due to the different nature of the consumed supplements (dietary versus performance supplements). According to a meta-analysis, however, due to the wide range of the prevalence of supplement intake among studies available, it is difficult to conclude whether the prevalence of dietary supplement use in athletes is due to a lack of homogeneity among studies [36]. In general, it appears that endurance athletes consume more supplements than athletes participating in sprint and short-distance activities [37,38]. Regarding the type of supplement, despite consistent data indicating multi-vitamins as the most consumed dietary supplement by elite athletes [33,34], there is some evidence demonstrating proteins [36] or creatine [39] are the most taken supplements by elite athletes. This contradiction can be simply attributed to the type and the nature of the sport being performed, and therefore, to the different nutritional requirements of the athletes [36].

When comparing vegan and non-vegan runners, a higher prevalence of supplement intake (irrespective of supplement type) was detected in vegans in the present study. While 72% of vegan runners reported consuming supplements, non-vegans reported consuming markedly fewer supplements (41% of omnivores and 35% of vegetarians). This occurrence might be directly linked to the increased consumption of vitamin supplements in vegans (66%), which was over two times higher than in omnivores and vegetarians (30% and 25%, respectively). However, this pronounced difference did not occur in either intake of CHO/protein or mineral supplements. The higher intake of vitamin supplements by vegan runners seems to be characterized by an increased health-consciousness of individuals who follow plant-based diets [16,21] and the existing dietary guidelines for vegan populations, primarily vegan athletes, in which supplementary intake of multi-vitamins is highly recommended [2,5,18]. According to the analysis of open questions in the present study, the average mineral intake was slightly lower in the vegan group than vegetarians and omnivores, which could be an alarming health concern for vegans who are at a higher risk of insufficient dietary intake of mineral nutrients. In a study comparing micronutrient status of vegan, vegetarian, and omnivorous endurance runners, all three groups consumed comparable frequencies of micronutrient supplements, except for vitamin B_12_, with a markedly higher consumption by vegan runners [19]. The consumption of vitamin B_12_ by vegan runners was noticeably higher than omnivorous and vegetarian runners in the present study, and recent evidence indicates that micronutrient deficiency can occur with any diet type [40]. Therefore, supplementation of critical nutrients including calcium, folate, magnesium, iron, copper, and vitamin D, vitamin B_12_–regardless of kind of diet–should be recommended for all groups of the public and athletic populations, and daily meals should be planned and composed appropriately [41]. In the present study, various micronutrients, macronutrients, and performance-enhancement substances (e.g., caffeine, alkaline salts, and nitrate) separately or in conjugation with other nutrients were reported by runners in open comments, but without further details to be expressively summarized. Therefore, as previously mentioned in another study on marathoners [35], caution is advised when interpreting the estimated frequencies of supplement intake due to the diverse nature of supplement products, which mostly contain added ingredients.

The present study showed that the prevalence of CHO/protein supplement intake was found to be similar among vegan (20%), vegetarian (18%), and omnivorous (20%) runners. While proteins and carbohydrates can play critical roles in health and performance separately, co-ingestion of carbohydrates and protein can result in positive effects on post-exercise recovery in endurance athletes, compared to carbohydrates alone [42], and particularly when applied in an appropriate ratio [43]. This was the main reason that CHO and protein supplements were combined in the analytical procedures of the present study. Notably, the well-documented CHO to protein ratio of 5:1 in vegans and 3:1 in omnivores [41,44] could generally provide optimal conditions for promoting health, performance, and recovery of vegan but not omnivorous athletes [1]. Nevertheless, considering the presence of undeclared ingredients in some protein supplements [45], athletes and their associated supporters should only consider supplements where a substantial body of scientific evidence proves their use as safe, legal, and effective [15].

### 4.2. Sex Differences

Sex-related differences in supplement intake have been investigated widely in athletes and sedentary individuals. Consistent with the previous literature, the results of the present study found no general difference between male and female athletes considering supplement intakes [36,41]. However, the limited number of contradictory data appears to be related to the type of supplements and/or to the characteristics of different kinds of sports. For example, males in bodybuilding [46] and females in track and field [47] are shown to use more supplements when compared to their counterparts of the opposite sex. Moreover, evidence shows that despite no overall sex-related difference in the frequency of supplement intake, male athletes were more likely to consume protein and ergogenic supplements associated with increased muscle mass, and female athletes were most likely to consume micro-nutrient supplements, typically associated with increased general health [36,39]. This account is partially but not entirely consistent with the present findings where females showed a higher prevalence in the use of vitamin (43% vs. 39%) and mineral (37% vs. 26%) supplements but not of CHO/protein (19% vs. 20%) than males. However, the non-significant sex-related differences in dietary supplement use may contribute to the nutritional concerns of both endurance athletes [35,38] and vegan/vegetarian populations [48], which involved 55% of participants in the present study.

### 4.3. Age Differences

Age is generally an important modulator of the decision to use supplements and the strongest predictor of dietary supplement intake [44,49]. The majority of studies investigating age differences in nutrient/supplement intake have compared young versus adult athletes [50,51]. In the present study, the wide range of age (18–66 years) allows an improved understanding of the age-related differences in supplement intake. In line with the present finding, reports from a study performed on team sports athletes showed that there was no association between age and supplement intake [52]. Inconsistently, numerous reports indicate a higher prevalence of nutritional supplement intake in senior athletes compared to juniors [49,50,51], which might be associated with the close association between age and the level of health-consciousness in athletes [49], or the higher frequent use of dietary supplements in elite athletes compared to their non-elite counterparts [36]. In this regard, a study found that the age-associated differences in supplement intake seemed to be linked with the level of performance, where 18-year-old athletes competing at the international level reported a higher supplement intake than younger athletes competing at national levels [50]. Recently, the higher prevalence of supplement intake among elite athletes was also emphasized by a review focusing on term professionalism in athletes [52].

### 4.4. Race Distance

In the present study, no significant difference was observed between 10-km runners, half-marathoners, (ultra-)marathoners, and the intakes of either CHO/protein, mineral, or vitamin supplements. Although no study has yet compared different groups of distance runners regarding the patterns of supplement intake, it has been well-documented that endurance athletes use supplements to a greater extent than non-endurance athletes [38,53], probably due to the higher exercise-induced nutrient requirements associated with long-time training, competition, and recovery [54]. In the present study, running and racing experiences had no association with the type of supplements taken by distance runners. This could be inconsistent with the previous reports indicating a 20% greater prevalence of supplement intake in athletes with a high training volume compared to athletes with a lower training volume [55]. Reports from a recent study on elite track and field athletes indicated that long-distance runners have a significantly higher prevalence in their use of both vitamin and mineral but not macronutrient supplements when compared to their counterparts in other track and field disciplines [56]. Moreover, in a study assessing the effects of vitamin and mineral supplement intake on ultra-marathon performance, it was found that the daily ingestion of multivitamin and mineral supplements, starting about one month before a competition, did not result in significant differences in performance time [57]. Despite considerable micronutrient requirements associated with endurance training and competition [6], there is insufficient evidence that multivitamin/multi-mineral supplements add benefits to long-distance runners, except for conditions where a clinical nutrient deficiency was diagnosed [54] and/or a competition-related adverse outcome (e.g., exercise-associated hyponatremia) occurs [58].

### 4.5. Macronutrient Distribution

The primary nutritional challenge that marathoners face is to meet the high exercise-induced requirements of daily energy and macronutrients necessary to optimize performance and recovery of prolonged training sessions [54,59]. In the present study, a higher intake of CHO (63% vs. 57% and 55%) along with a lower protein (22% vs. 24% and 26%) and fat (15% vs. 19% and 19%) intake was reported by vegan runners compared to vegetarian and omnivore runners, respectively (data are expressed as a percentage of total daily calories). Due to the lack of discrimination between vegan and vegetarian kinds of diet in the majority of most similar studies, it is difficult to compare and interpret the present results with the findings of the previously related investigations. According to the literature available, vegetarians (including vegans when pooled) have been previously reported to consume a higher daily energy intake from carbohydrates (51.4–69.1%), less protein (8.2–13.5%), and less fat (18.4–36.2%) compared to omnivores [41,44,60]. Inconsistently, however, one study found no significant difference in carbohydrate intake between sedentary vegetarian and omnivorous women [23]. 

Although it is well-accepted that individualized macronutrients distribution depends on different training, individual, nutritional, and environmental characteristics, macronutrient distribution of 60% from CHO, 15% from protein, and 25% from fat have been recommended in general to support repeated bouts of endurance training and to meet recommendations for health concerns [29,54]. The present findings showed that only vegan endurance runners met the recommendations for CHO intake by 63% of their total daily energy intake, which might be due to the characteristics of the study population involving 55% vegans and vegetarians. A greater proportion of carbohydrates, however, may warrant ultra-endurance runners’ needs [16]. Consistent with the results of vegan runners from the present study, East African distance runners who supply 60–80% of their energy needs from carbohydrates, interestingly have 80–90% reliance on plant-based foods [61,62]. More remarkably, another example is a mountain biker who successfully and healthfully completed an ultra-endurance stage event with a well-planned vegan diet involving 83% CHO [13]. 

In the present study, protein intakes of vegan, vegetarian, and omnivorous runners were higher than the recommended range for endurance athletes [63], which was found to be at the expense of dietary fat intake with lower values than recommended for endurance athletes [16,64]. This might be explained by the nutritional concerns of both distance runners and vegetarians, but especially the vegan individuals are emphasized to pay careful attention to adequate protein consumption [2,5,41], along with their higher level of health consciousness [21]. Additionally, misreporting of dietary protein and fat intake, which is somewhat more prevalent in athletic populations [65], could be another justification. As a practical recommendation, however, consumption of different groups of plant-based foods is advised to vegan and vegetarian athletes to meet nutritional guidelines for optimizing health and performance [2,41].

### 4.6. Limitations and Strengths 

There are some limitations to this study to be mentioned. In addition to the relatively small sample size, the study was conducted following a cross-sectional design, and the findings are based on self-reported data. Therefore, the results have to be interpreted with caution. In order to minimize the effect and to control for inconsistent and contradictory statements, control questions were implemented in different sections of the questionnaire. Moreover, despite the higher proportion of vegan/vegetarian populations in DACH countries (10–14%) compared to other Western nations, this might have affected the present results to some extent as 55% of participants in the present study stated following a vegan or vegetarian diet, which is markedly higher than the worldwide numbers, as well as for German-speaking countries (potential selection bias). Another limitation of the present study could be the impossibility of conducting statistical analysis for inconsistent and incomplete open comments provided by runners, which compelled the authors to report the relevant findings inclusively. However, self-reports for this type of variable are valid if they are collected immediately or shortly after an event [66]. In this study, the average time between completion of the last event and completion of the questionnaire by the participants was unknown (see the fifth inclusion criteria): self-reports refer to at least one adequate running event completed within the past two years). Therefore, the validity of the self-report of the current study is unknown and not applicable.

Nevertheless, the authors are convinced that the present findings contribute to adding a valuable and novel body of evidence considering patterns of dietary supplement intake among endurance runners with a critical focus on runners who follow plant-based diets. Additionally, the present findings may help future investigations by design to identify specific requirements of endurance runners when adhering to specific kinds of diet. Future research with large randomized samples of distance runners can add support in providing comparable data on patterns of supplement intakes to help meet the macronutrient and micronutrient intake guidelines, which would especially contribute to a better understanding of supplement intake in distance runners following plant-based dietary patterns.

## 5. Conclusions

In summary, the present comparison of vegan, vegetarian, and omnivorous distance runners shows a more than two-fold higher intake of vitamin supplements, but not of mineral and CHO/protein supplements, among vegan versus non-vegan runners. This finding might be associated with the existing dietary guidelines that highly recommend the supplementary intake of critical micronutrients (e.g., vitamin B_12_, vitamin D, calcium, iron, iodine, zinc) for vegan athletes. No difference between 10-km runners, half-marathoners, and (ultra-)marathoners in the type of consumed supplements were observed, indicating the null effects of the race distance on supplement choice among long-distance runners. Overall, the findings from the present study point to an important contribution of supplement strategies in endurance runners, particularly those who follow a vegan diet, and might be helpful when applying personalized nutritional strategies for optimized individual health status, performance, and recovery.

## Figures and Tables

**Table 1:** Descriptive characteristics of participants displayed by dietary subgroups.

		Total *n* = 220	Omnivorous *n* = 100	Vegetarian *n* = 40	Vegan *n* = 80
**Age (years)**		38.5 (IQR 18)	43 (IQR 16.5)	37.5 (IQR 16)	36 (IQR 14.25)
**Body Weight (kg)**		65.0 (IQR 14.4)	68.4 (IQR 15.8)	61 (IQR 8.1)	64.1 (IQR 11)
**Height (m)**		1.7 (IQR 0.1)	1.7 (IQR 0.1)	1.7 (IQR 0.1)	1.7 (IQR 0.1)
**BMI (kg/m^2^)**		21.75 (IQR 3.44)	22.54 (IQR 3.55)	20.75 (IQR 3.4)	21.32 (IQR 3)
**Sex**	Female	127 (58%)	47 (47%)	26 (65%)	54 (68%)
	Male	93 (42%)	53 (53%)	14 (35%)	26 (32%)
**Race Distance**	10 km	79 (36%)	35 (35%)	12 (30%)	32 (40%)
	Half marathon	84 (38%)	37 (37%)	19 (48%)	28 (35%)
	Marathon/Ultra-marathon	57 (26%)	28 (28%)	9 (22%)	20 (25%)
**Academic** **Qualification**	No qualification	1 (<1%)	0 (0%)	0 (0%)	1 (1%)
	Upper secondary	72 (33%)	36 (36%)	16 (40%)	20 (25%)
	Education/Technical				
	A Levels or equivalent	52 (24%)	26 (26%)	7 (18%)	19 (24%)
	University/Higher degree	73 (33%)	31 (31%)	13 (32%)	29 (36%)
	No answer	22 (10%)	7 (7%)	4 (10%)	11 (14%)
**Marital Status**	Divorced/Separated	11 (5%)	3 (3%)	1 (2%)	7 (9%)
	Married/Living with partner	149 (68%)	75 (75%)	23 (57%)	51 (64%)
	Single	60 (27%)	22 (22%)	16 (40%)	22 (28%)
**Country of Residence**	Austria	39 (18%)	21 (21%)	6 (15%)	12 (15%)
	Germany	161 (73%)	71 (71%)	32 (80%)	58 (72%)
	Switzerland	11 (5%)	7 (7%)	1 (2%)	3 (4%)
	Other	9 (4%)	1 (1%)	1 (2%)	7 (9%)

BMI—body mass index. IQR—interquartile range.

**Table 2:** Prevalence of self-reported macronutrient intake, supplement intake, and most frequently used micronutrient supplements from minerals and vitamins.

		Total (*n* = 121)	Omnivorous (*n* = 40)	Vegetarian (*n* = 16)	Vegan (*n* = 65)
**Macronutrients**	CHO	58.2%	55%	57%	63%
	Protein	24.5%	26%	24%	22%
	Fat	17.4%	19%	19%	15%
**Supplement** **Consumption**	CHO/Protein	43 (20%)	20 (20%)	7 (18%)	16 (20%)
	Minerals	72 (33%)	36 (36%)	9 (22%)	27 (33%)
	Vitamins	93 (42%)	31 (30%)	10 (25%)	52 (66%)
	Other supplements	13 (6%)	6 (6%)	1 (2%)	6 (8%)
**Minerals**	Calcium	5%	8%	13%	2%
	Iron	11%	13%	13%	9%
	Magnesium	28%	45%	25%	18%
	Zinc	12%	13%	6%	6%
**Vitamins**	Folate	4%	3%	13%	3%
	Vitamin B12	35%	5%	38%	52%
	Vitamin C	6%	13%	-	3%
	Vitamin D	12%	18%	-	12%
	Vitamin B-complex	7%	8%	-	8%
	Multivitamin	28%	30%	19%	29%

CHO—carbohydrates.

**Table 3:** Effects of sex, age, diet type, and race distance on supplement intake.

		CHO/Protein			Minerals			Vitamins		
		Fit	CI	*p*	Fit	CI	*p*	Fit	CI	*p*
**Sex**	Female	0.19	[0.13, 0.27]	0.879	0.37	[0.29, 0.46]	0.118	0.43	[0.34, 0.53]	0.642
	Male	0.20	[0.13, 0.30]		0.26	[0.18, 0.37]		0.39	[0.29, 0.51]	
**Age (y)**	20	0.21	[0.12, 0.35]	0.713	0.26	[0.16, 0.40]	0.277	0.33	[0.21, 0.47]	0.158
	30	0.20	[0.14, 0.28]		0.29	[0.22, 0.38]		0.37	[0.28, 0.47]	
	40	0.19	[0.15, 0.25]		0.32	[0.26, 0.39]		0.42	[0.35, 0.49]	
	50	0.18	[0.12, 0.27]		0.36	[0.27, 0.45]		0.47	[0.37, 0.57]	
	70	0.17	[0.07, 0.37]		0.43	[0.24, 0.64]		0.57	[0.35, 0.76]	
**Diet Type**	Omnivorous	0.20	[0.13, 0.29]	0.951	0.36	[0.27, 0.46]	0.266	0.30	[0.22, 0.40]	0.000
	Vegetarian	0.18	[0.09, 0.33]		0.22	[0.12, 0.38]		0.25	[0.14, 0.41]	
	Vegan	0.20	[0.12, 0.30]		0.33	[0.23, 0.44]		0.66 *	[0.54, 0.75]	
**Race Distance**	10 km	0.19	[0.12, 0.29]	0.768	0.34	[0.25, 0.46]	0.571	0.46	[0.35, 0.59]	0.613
	HM	0.18	[0.11, 0.28]		0.28	[0.19, 0.39]		0.39	[0.28, 0.50]	
	M/UM	0.23	[0.13, 0.36]		0.35	[0.24, 0.49]		0.39	[0.26, 0.53]	

* Significant differences within groups. CHO—carbohydrates. 10 km—10-km. HM—half-marathon. M/UM–marathon/ultra-marathon. CI—mean effect size with 95%-CI, upper and lower boundaries. *P*—*p*-value.

## Data Availability

The datasets generated during and/or analyzed during the current study are not publicly available but may be made available upon reasonable request. Subjects will receive a brief summary of the results of the NURMI Study if desired.
